# Investigation of the thermal tolerance of silicon-based lateral spin valves

**DOI:** 10.1038/s41598-021-90114-9

**Published:** 2021-05-19

**Authors:** N. Yamashita, S. Lee, R. Ohshima, E. Shigematsu, H. Koike, Y. Suzuki, S. Miwa, M. Goto, Y. Ando, M. Shiraishi

**Affiliations:** 1 Department of Electronic Science and Engineering, Kyoto University, Kyoto, Kyoto 615-8510 Japan; 2 Advanced Products Development Center, TDK Corporation, Ichikawa, Chiba 272-8558 Japan; 3 Graduate School of Engineering Science, Osaka University, Toyonaka, Osaka 560-8531 Japan; 4 Institute for Solid State Physics, The University of Tokyo, Kashiwa, Chiba 277-8581 Japan

**Keywords:** Materials for devices, Electrical and electronic engineering, Nanoscale devices

## Abstract

Improvement in the thermal tolerance of Si-based spin devices is realized by employing thermally stable nonmagnetic (NM) electrodes. For Au/Ta/Al electrodes, intermixing between Al atoms and Au atoms occurs at approximately 300 °C, resulting in the formation of a Au/Si interface. The Au–Si liquid phase is formed and diffuses mainly along an in-plane direction between the Si and AlN capping layers, eventually breaking the MgO layer of the ferromagnetic (FM) metal/MgO electrodes, which is located 7 µm away from the NM electrodes. By changing the layer structure of the NM electrode from Au/Ta/Al to Au/Ta, the thermal tolerance is clearly enhanced. Clear spin transport signals are obtained even after annealing at 400 °C. To investigate the effects of Mg insertion in FM electrodes on thermal tolerance, we also compare the thermal tolerance among Fe/Co/MgO, Fe/Co/Mg/MgO and Fe/Co/MgO/Mg contacts. Although a highly efficient spin injection has been reported by insertion of a thin Mg layer below or above the MgO layer, these thermal tolerances decrease obviously.

## Introduction

Silicon (Si) is a suitable material for semiconductor-based spintronic devices because of its long spin lifetime^1,2^, excellent controllability of spin current by gate voltage^3,4^, and availability of mature infrastructures of Si-based electronic devices. Significant advances, including room-temperature demonstrations of Si-based spin devices such as a spin metal-oxide semiconductor field-effect transistor^4–9^ and a magneto logic gate^10,11^, have been achieved to date by using a magnesium oxide (MgO) tunnel barrier. Recent studies have clarified the spin scattering mechanism in Si based on Elliott–Yafet mechanisms^2,12,13^, and optical phonon emissions suppress the spin diffusion length under high electric fields with applied gate voltage^3^. A remaining problem for the practical application of spin devices is a small signal amplitude in transistor and logic operations due to an insufficient spin accumulation voltage in Si. Thus, exploring a ferromagnetic contact enabling a large spin accumulation voltage in Si is a significant research issue, and half-metallic materials^14–16^ and the insertion of a thin magnesium (Mg) layer^9,17–20^ in a ferromagnetic contact in Si spin devices have been extensively investigated. Among these methods, the insertion of a thin Mg layer above/below the MgO layer is a new approach to enhancing spin signals in spin devices. Indeed, the insertion of the Mg layer above the MgO layer allows for the enhancement of the spin polarization of Fe on Mg/MgO^9,17,18^ due to suppression of the formation of a magnetic dead layer, and the insertion of Mg below the MgO layer provides a highly textured MgO tunnelling barrier on the Si channel, which enhances the magnitude of spin signals^20^.

Recently, we established and reported another way to increase spin signals in nondegenerate Si-based spin devices, which involves thermal annealing the Si spin devices at 300 °C, and a twofold increase in spin accumulation voltage was realized^21^. This enhancement was attributed to improved crystallinity in the MgO tunnelling barrier and better spin polarization. However, no spin accumulation signals were detected in that study after annealing above 300 °C^21^, which presents a new problem for practical applications. The thermal tolerance of Si spin devices at least above 350 °C is strongly desired to obtain compatibility with the infrastructures of Si-based electronic devices since the post fabrication process often employs thermal treatments above 300 °C^22–24^. Therefore, it is significant to clarify the degradation mechanism of Si spin devices by thermal annealing and to solve the problem of further progress in Si spintronics.

Here, we have investigated the thermal degradation mechanisms of a lateral spin valve (LSV) based on nondegenerate n-type Si by electric and crystallographic methods. It is revealed that the in-plane long-range diffusion of gold (Au) atoms in nonmagnetic (NM) electrodes occurs through the aluminium nitride (AlN)/Si interface. The Au atoms eventually reach a ferromagnetic (FM) electrode that is located 7 µm away from the NM electrodes, resulting in the degradation of the MgO tunnelling barrier. To improve the thermal tolerance, the layer structure of the NM electrode is changed from Au/Ta/Al to Au/Ta. The modified device clearly exhibits spin signals even after annealing at 400 °C. We also focus on the thermal tolerance of different structures in FM contacts without Mg insertion and Mg insertion below/above the MgO layer, as enhancement of the spin polarization has been reported by insertion of a thin Mg layer^17,18,20^. However, the thermal tolerance obviously decreases down to approximately 300 °C due to the existence of the Mg layer.

## Results and discussion

**Figure 1:**
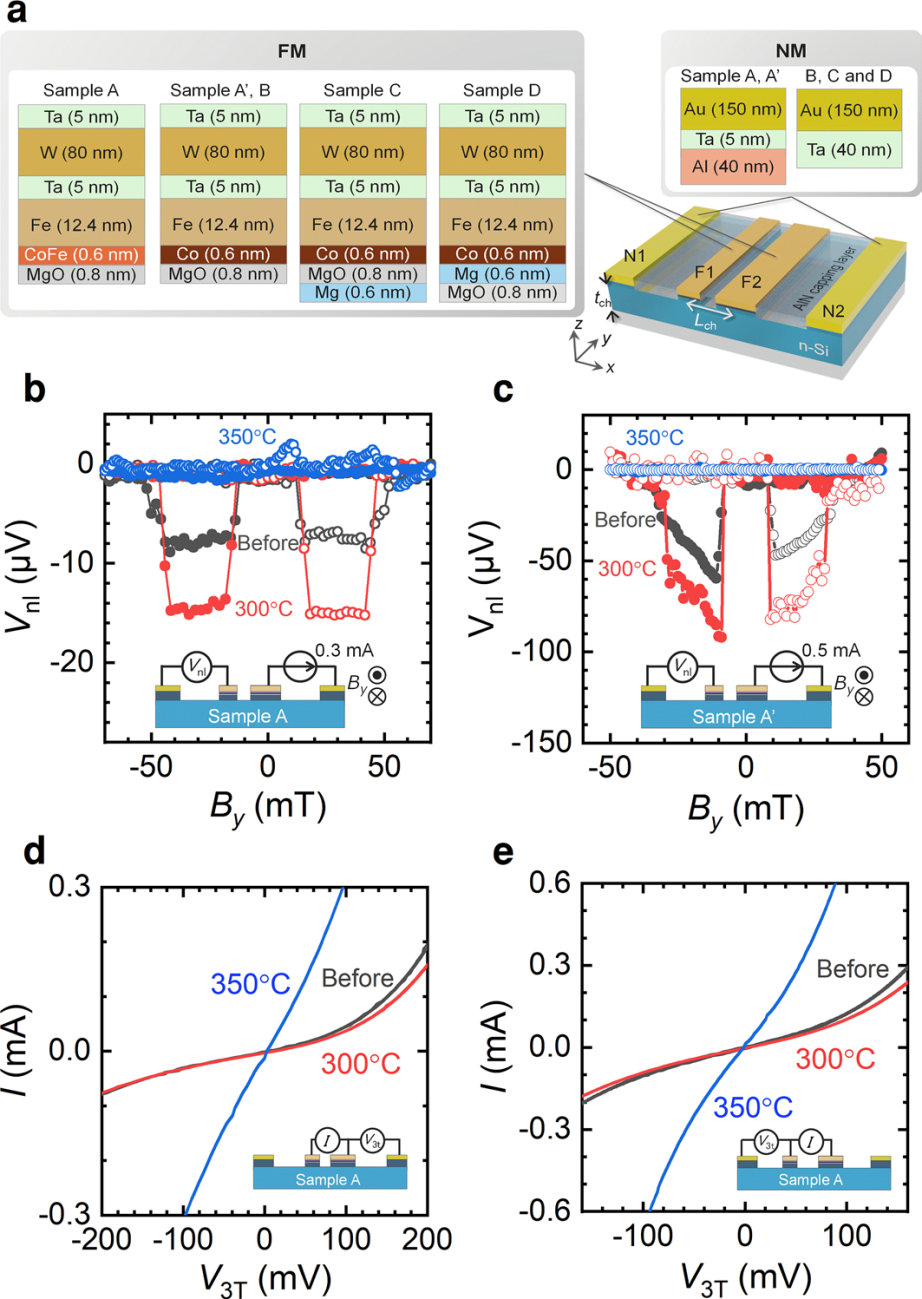
(**a**) A schematic of Si-based lateral spin valves. The channel width along *y* direction was 21 µm. (**b**) Spin accumulation signals, i.e., nonlocal voltage *V*_nl_ as a function of magnetic flux density along the *y* direction *B*_*y*_, for sample A before annealing (black) and after annealing at 300 °C (red) and 350 °C (blue), obtained by the NL4T method. The channel length *L*_ch_ was 2.0 µm, and the channel thickness *t* was 80 nm. The widths of F1 and F2 were 0.2 and 1.2 µm, respectively. (**c**) Spin accumulation signals of sample A’ before annealing (black) and after annealing at 300 °C (red) and 350 °C (blue), obtained by the NL4T method. The channel length *L*_ch_ was 1.6 µm, and the channel thickness *t* was 100 nm. The widths of F1 and F2 were 0.5 and 2.0 µm, respectively. (**d**, **e**) *I*–*V* characteristics of (**d**) F1 and (**e**) F2 before annealing (black) and after annealing at 300 °C (red) and 350 °C (blue) for sample A.

Schematics of the device geometry and layer structure of the Si-based LSVs are shown in Fig. 1a. The devices consist of n-type Si, two FM electrodes (F1 and F2), two NM electrodes (N1 and N2) and an AlN capping layer on the Si channel. The details of sample preparation are described in the Methods section. The spin transport properties, such as the spin polarization and the spin diffusion length, were measured by nonlocal four-terminal (NL4T)^25^ and local three-terminal (L3T)^26^ measurements at room temperature. Crystallographic analyses of the devices were carried out by means of cross-sectional transmission electron microscopy (TEM) with energy dispersive X-ray spectroscopy (EDS).

First, sample A was employed to investigate the thermal tolerance and degradation mechanism of the spin transport properties. The spin signals were measured by means of NL4T measurements, and the *I*–*V* characteristics of F1 and F2 are measured at 300 K. Spin accumulation signals, i.e., a nonlocal voltage *V*_nl_, as a function of *B*_*y*_ before and after thermal annealing, are shown in Fig. 1b. A clear rectangular shape was measured, indicating successful spin transport. The magnitude of the spin accumulation signals was increased after annealing at 300 °C, consistent with our previous report^21^. In contrast, the spin accumulation signal completely disappeared after annealing at 350 °C. These results were observed in both devices using CoFe (Fe/CoFe/MgO/Si) and Co (Fe/Co/MgO/Si) as shown in Fig. 1c, which implies that the heat tolerance of the FM electrodes does not affect the results, i.e., that of the NM electrodes governs the tolerance. To investigate the electrical properties of FM1 and FM2, three terminal *I*–*V* characteristics are measured. Figure 1d, e exhibit the three terminal *I*–*V* characteristics of F1 and F2 before/after annealing. Whereas the *I*–*V* characteristics did not change after annealing at 300 °C, they decreased significantly after annealing at 350 °C, which was consistent with our previous study^21^. The reduction in the spin signal is attributable to the reduction in the interfacial resistance from 1.5 × 10^−8^ to 1.6 × 10^−9^ Ωm^2^ in F1 and from 1.5 × 10^−8^ to 4.5 × 10^−9^ Ωm^2^ in F2 due to the conductance mismatch^21,27^. The one-dimensional spin diffusion model (see methods) also revealed that the spin polarization decreases from approximately 2.8% to less than 1.3% after annealing at 350 °C.

**Figure 2:**
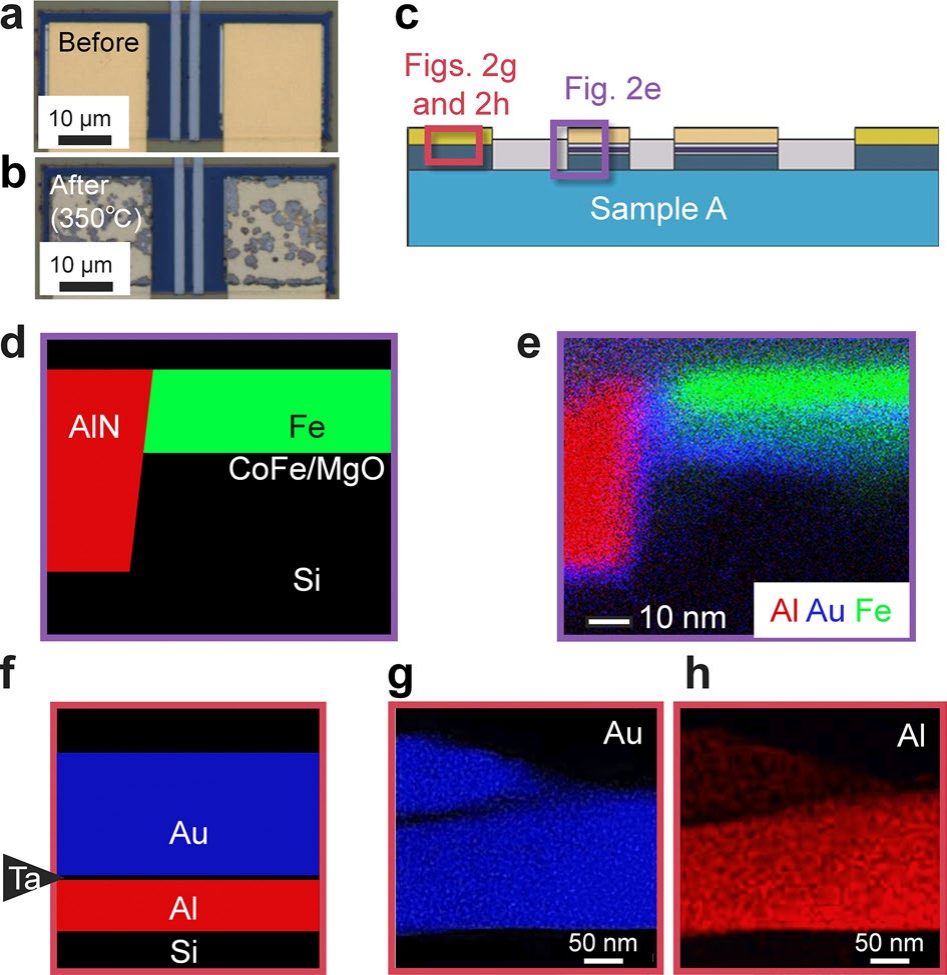
Microscopic pictures of sample A after annealing at 350 °C. (**a**, **b**) Optical microscopic image (**a**) before and (**b**) after annealing at 350 °C. (**c**) A schematic cross-section of sample A. The areas of EDS observations are roughly indicated by purple and red squares. (**d**) A designed structure and (**e**) an elemental mapping image near the FM electrode indicated by the purple rectangle in (c), where Au, Al and Fe are shown in blue, red and green, respectively. (**f**) Designed structure and **e**lemental mapping images of (**g**) Au and (**h**) Al near the NM electrode indicated by the red rectangle in (c).

**Figure 3:**
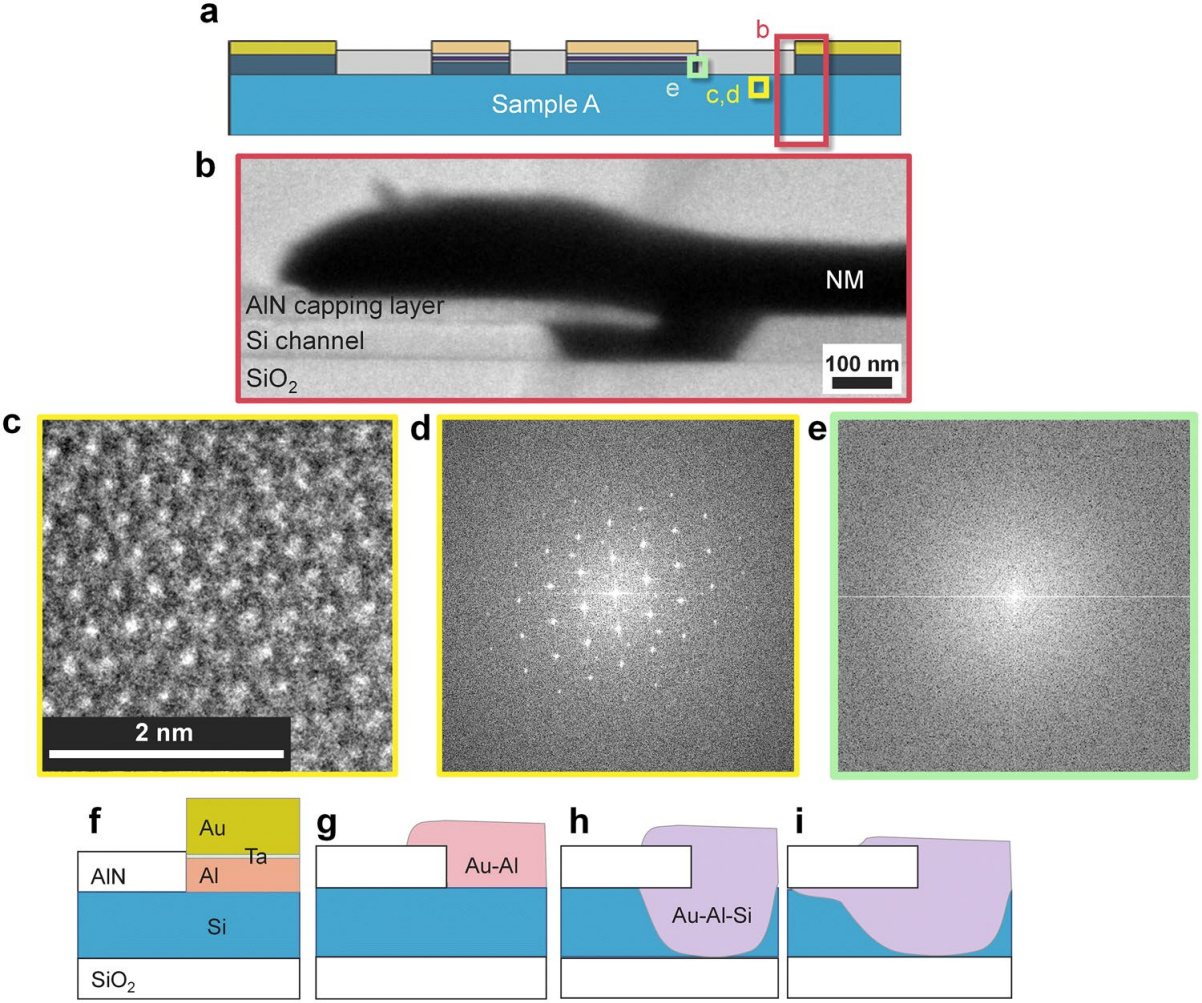
Crystallographic analyses of the Si channel after annealing and a possible degradation mechanism by annealing. (**a**) A schematic cross-section of sample A. The areas of TEM observations are roughly indicated by red and yellow squares. (**b**) A cross-sectional TEM image of the NM electrode obtained at the red square shown in (**a**). (**c**) An atomic-resolution TEM image obtained at the Si channel indicated by the yellow rectangle in (**a**) and (**d**) the Fourier transformation of the area. (**e**) A Fourier transformation of the area of Au diffusing along AlN/Si interface. (**f**)**–**(**i**) Schematic images of the possible mechanism of diffusion of the Au atoms during annealing. (**f**) the fabricated structure. (**g**) Au-Al intermetallics were formed, and Au reached Si. (**h**) The intermetal of Au-Al-Si was formed, including the liquid phase. (**i**) The liquid phase diffused through the interface of AlN/Si.

Figure 2a, b show optical microscopy images of sample A before annealing and after annealing at 350 °C. Although a smooth gold surface was confirmed at the NM electrodes before annealing, a patchy pattern in gold, grey and blue was apparent after annealing. In contrast, no significant changes were observed on the surface of the FM electrodes. To reveal the degradation mechanism in detail, crystallographic analyses using TEM and EDS were carried out. A cross-sectional elemental mapping images obtained by EDS are shown in Fig. 2e, g, h. We examined the cross-section of the FM electrodes indicated by the purple square in Fig. 2c. While Au was not used in FM electrodes (Fig. 2d), Au atoms, shown in blue, migrate from the NM electrode and were found around the FM electrodes (Fig. 2e). These electrodes were separated 7 µm away from each other. Then, we examined an NM electrode consisting of an Au/Ta/Al trilayer (Fig. 2f) using the cross-sectional elemental mappings shown in Fig. 2g, h, revealing that Au atoms diffused in the Al layer and reach the Al/Si interface. TEM observation was carried out to investigate the crystallographic properties of the NM/Si interface and the Si channel to trace the path of Au diffusion. We observed the areas indicated in Fig. 3a. Whereas intermixing of Si channels and Au atoms was recognized beneath the NM electrodes, as shown in Fig. 3b, the intermixing area was limited only beneath the NM electrode. Figure 3c, d show atomic-resolution TEM images and the Fourier transformation obtains a part of the [110] surface of the Si channel ca. 0.4 µm away from the NM electrodes. The Fourier transformation shows hexagonal spot patterns, which is evident in the clear periodicity of the diamond structure, indicating no significant structural damage to the Si channel. On the contrary, the Au diffusing area along the AlN/Si interface shows a halo feature (Fig. 3e), indicating a significant degradation of the crystal structure. Since the Au–Si system exhibits the eutectic alloys with a considerably low solubility limit of Au atoms in the Si phase, a segregation of Au atoms is expected if the Si channel dominantly contributes the Au diffusion. Because no Au particles were confirmed near the bottom side of the Si layer, the Si channel is not a probable diffusion path for the Au atoms.

**Figure 4:**
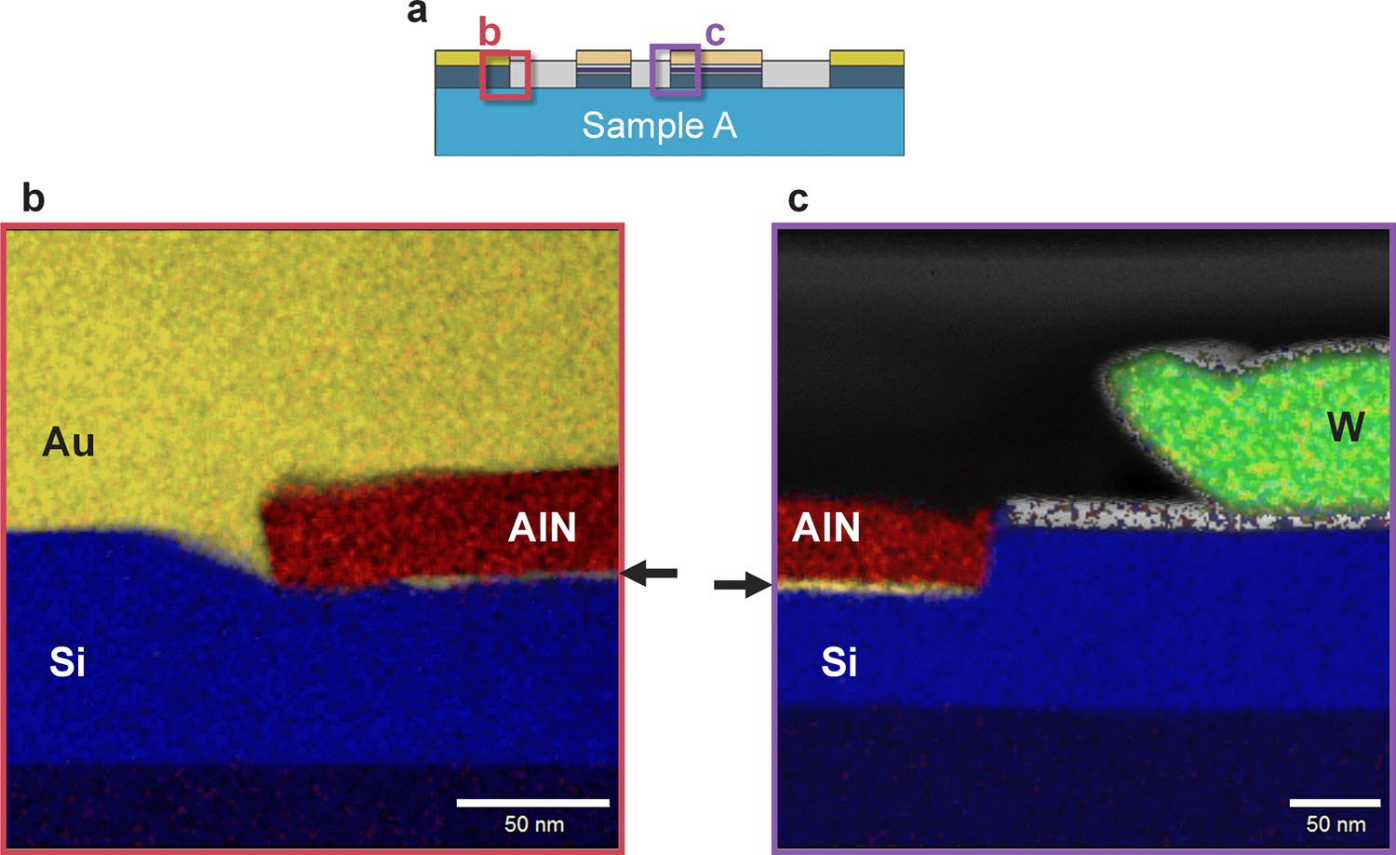
Elemental mapping images of other parts of sample A. (**a**) A schematic cross-section of sample A. The examined areas are indicated by the red (**b**) and the purple (**c**) rectangles. (**b**) The overlay of the elemental mapping around N1. The blue, yellow, and red area indicate Si, Au, and N near the NM electrode. Au was detected from the AlN/Si interface, which is indicated by the arrow. (**c**) The overlay of the elemental mapping around F2. The blue, yellow, red, and green area indicate Si, Au, N, and W near the FM electrode. Au was detected from the AlN/Si interface, which is indicated by the arrow.

We then clarify the possible mechanism of Au atom diffusion by annealing. The Al and Si binary systems exhibit eutectic alloys, but the temperature of the eutectic point is above 570 °C, indicating that the Al-Si liquid phase is not formed at the Al/Si interface during the annealing process. Similarly, Ta-Si, Ta-Al and Au-Al systems are expected to maintain their solid states below 400 °C. In contrast, the temperature of the eutectic point of the Au–Si system is approximately 350 °C. Considering the above situations, a possible mechanism of Au atom diffusion is as follows: The designed structure is shown in Fig. 3f. First, the intermixing of Al and Au atoms takes place below 300 °C, as schematically shown in Fig. 3g, which is confirmed by EDS (Fig. 2g, h) despite the 5-nm Ta layer. The interdiffusion length *l* of Au and Al atoms, estimated from $l = 5.56 \times 10^{ - 10} t$ m at 350 °C^28^, is 2 µm after *t* = 1 h. Therefore, Au atoms easily penetrate the Al layer, which is only 40 nm in thickness, and finally reach the Si channel. Since the melting point of Au_8_Si_2_ is approximately 350 °C, Au–Si and/or Au-Al-Si liquid phases are formed at the Au–Si interface (Fig. 3h). Because Au_8_Si_2_ is the eutectic point, additional Au and Si atoms rapidly increase the melting point, so most of the Si channel remains without reaction. The Au–Si and/or Au-Al-Si liquid phases diffuse mainly along the AlN/Si interface (Fig. 3i) due to the strong tensile strain^29^, and the interface is more susceptible to destruction than the other areas. Finally, Au reaches the FM electrodes and invades the MgO tunnelling barrier. To confirm the repeatability, elemental mapping images of other parts at the AlN/Si interface of sample A were also obtained around the NM and FM electrodes as shown in Fig. 4. Au was detected at the AlN/Si interface even between the FM 1 and 2, i.e., a part of the spin transport channel as indicated by the arrows in Fig. 4b, c. Therefore, not only FM contacts but also the spin transport channel was damaged by the Au atoms.

**Figure 5:**
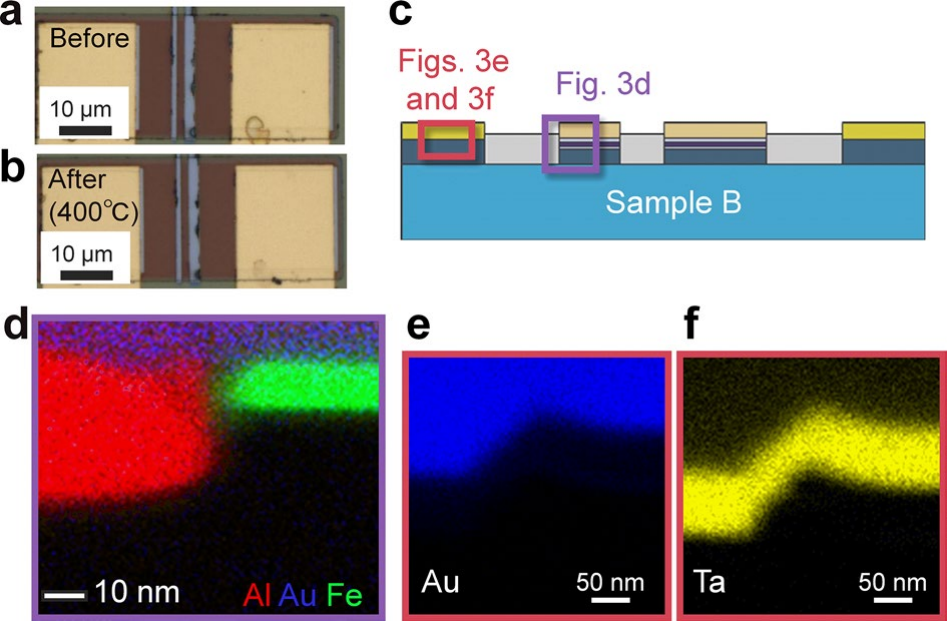
Microscopic pictures of sample B after annealing at 400 °C. (**a**, **b**) Optical microscopic image (**a**) before and (**b**) after annealing at 400 °C. (**c**) A schematic cross-section of sample B. The areas of EDS observations are roughly indicated by purple and red squares. (**d**) Elemental mapping image near the FM electrode indicated by the purple rectangle in (c), where Al, Au and Fe are shown in red, blue and green, respectively. (**e**, **f**) Elemental mapping images of (**e**) Au and (**f**) Ta near the NM electrode indicated by the red rectangle in (c).

To confirm the thermal tolerance of FM electrodes, a control experiment was performed using sample B. To circumvent the interdiffusion of Al and Au, we excluded Al from the NM electrodes and employed a thick Ta layer: Au(150 nm)/Ta(40 nm). Ta is often used as a barrier metal for Si against conductive metals such as Cu because the Ta layer is maintained even after annealing at 630 °C, which is high enough for any thermal treatments applied to electronic devices^30^. Thermal annealing at 300 °C, 350 °C, and 400 °C was carried out. The optical microscope image of sample B after annealing at 400 °C showed no salient changes on the surface of the Au/Ta electrode (see Fig. 5a, b), which was in contrast to the Au/Ta/Al structure in sample A. Cross-sectional elemental mapping images (see Fig. 5c indicating the examined parts) showed no signal from Au atoms detected at the AlN/Si interface or around FM even after annealing at 400 °C (see Fig. 5d). Furthermore, the NM electrode clearly maintains the Ta/Au bilayer structure (see Fig. 5e, f). These results are obviously different from those of sample A, which directly supports our hypothesis of the thermal degradation of a Si-based LSV.

**Figure 6:**
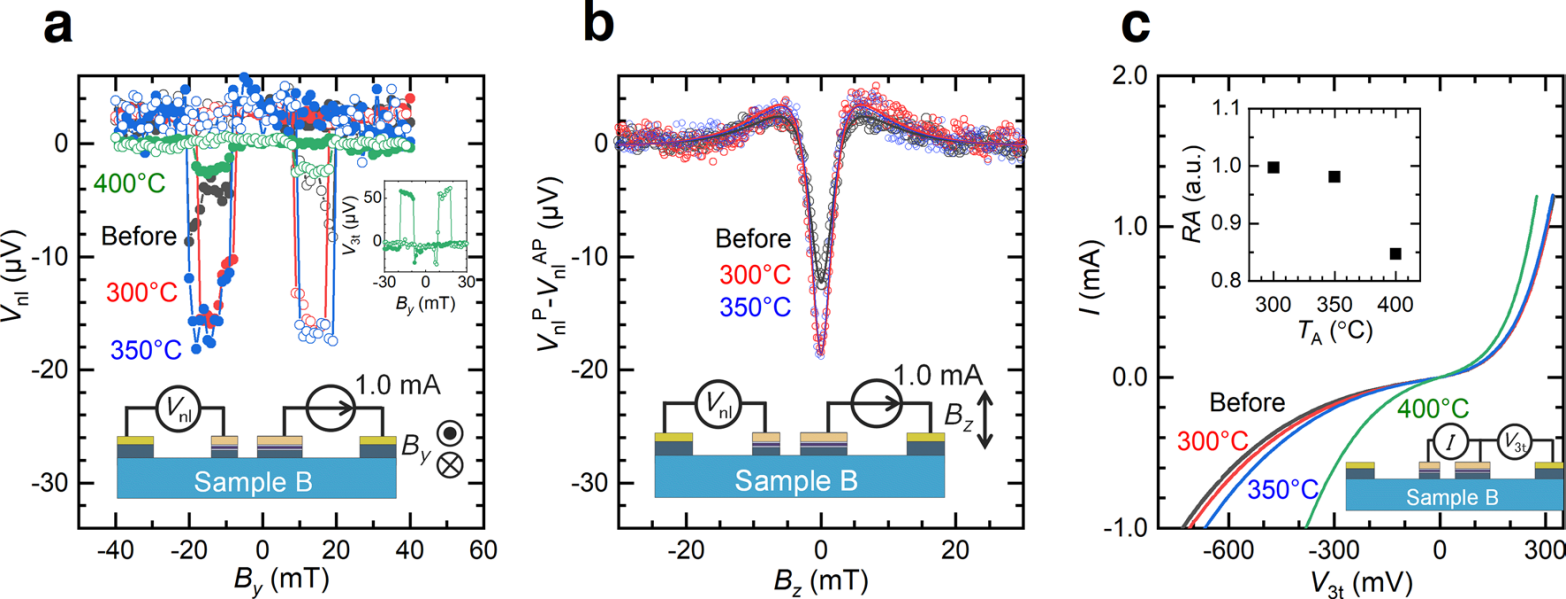
Results of the control experiments with sample B, which has NM electrodes made of Au/Ta. *L*_ch_ = 2.75 µm, *t* = 100 nm for sample B. The widths of F1 and F2 were 0.5 and 2.0 µm, respectively. (**a**) The spin accumulation signals of sample B measured by the NL4T method. Black, red, blue and green show the measured *V*_nl_ before annealing after annealing at 300 °C, 350 °C and 400 °C, respectively. The inset shows the result of the L3T measurement after annealing at 400 °C. (**b**) Results of the Hanle measurements of sample B. Black, red and blue show the measured *V*_nl_ before annealing after annealing at 300 °C and 350 °C, respectively. (**c**) *I*–*V* characteristics of sample B. Black, red, blue and green show the measured *V*_nl_ before annealing after annealing at 300 °C, 350 °C and 400 °C, respectively. The inset shows the *RA* product normalized by the value before annealing measured at 1.0 mA.

To confirm the thermal tolerance of the LSV, NL4T measurements were carried out. Clear hysteresis features were obtained for all conditions, i.e., before annealing and after annealing at 300, 350 and 400 °C, as shown in Fig. 6a. The magnitude of the spin accumulation signal before annealing was 9 µV, and the signal was enhanced to 18 µV after annealing at 300 °C, similar to that of sample A. In contrast to sample A, the spin signal did not decrease after annealing at 350 °C. Furthermore, clear spin accumulation signals were also obtained even after annealing at 400 °C. To corroborate the tolerance of the spin diffusion length, Hanle signals were also measured in the NL4T method for sample B after annealing at 300 and 350 °C, as shown in Fig. 6b. To eliminate the spurious effects, the difference in *V*_nl_ between the parallel and antiparallel magnetic configurations, $V_{{{\text{nl}}}}^{{\text{P}}} - V_{{{\text{nl}}}}^{{{\text{AP}}}}$, was employed for the analyses. The spin diffusion length of the Si channel *λ* was estimated from the curve fitting by using the following equation^13^:

1 $$ V_{{{\text{nl}}}}^{{\text{P}}} \left( {B_{z} } \right) - V_{{{\text{nl}}}}^{{{\text{AP}}}} \left( {B_{z} } \right) = \pm S_{0} \mathop \smallint \limits_{0}^{\infty } \sqrt {\frac{1}{4\pi Dt}} {\text{cos}}\left( {\omega t} \right){\text{exp}}\left( { - \frac{t}{\tau }} \right)\exp \left( { - \frac{{L_{{{\text{ch}}}}^{2} }}{4Dt}} \right)dt, $$

where *B*_*z*_ is the magnetic flux density along the *z*-direction, *S*_0_ is a constant value that determines the magnitude of the signal, *D* is the diffusion constant, *ω* = *gµ*_B_*B*_*z*_*/ℏ* is the Larmor frequency, *g* = 2 is the *g* factor for the electrons, *µ*_B_ is the Bohr magneton, *ℏ* is the Dirac constant and *τ* is the spin life time. The fitting curves obtained using Eq. (1) reproduced the experimental results well, as shown in Fig. 6b. $\lambda = \sqrt {D\tau }$ was 1.6 ± 0.1 µm before annealing, 1.7 ± 0.1 µm after annealing at 300 °C, and 1.8 ± 0.1 µm after annealing at 350 °C. No significant changes in spin diffusion length were confirmed after annealing below 350 °C, as expected. Because the magnitude of the spin accumulation voltage was considerably small, the precise estimation of $\lambda$ after annealing at 400 °C was difficult. In fact, the interfacial resistance obviously decreased after annealing at 400 °C (see Fig. 6c), and the FM electrode degraded. However, identical characteristics were observed before annealing and after annealing at 300 °C and 350 °C. The inset shows the resistance area products *RA* normalized by the value before annealing. *RA* slightly decreased after annealing at 350 °C and steeply decreased to 0.85 after annealing at 400 °C, which is similar trend as observed in the previous study^16^. Because nonlinear *I*–*V* characteristics are maintained, the condition of the MgO barrier is expected to be different from that of sample A, which might reflect an inherent thermal tolerance of the Fe/Co/MgO contact.

**Figure 7:**
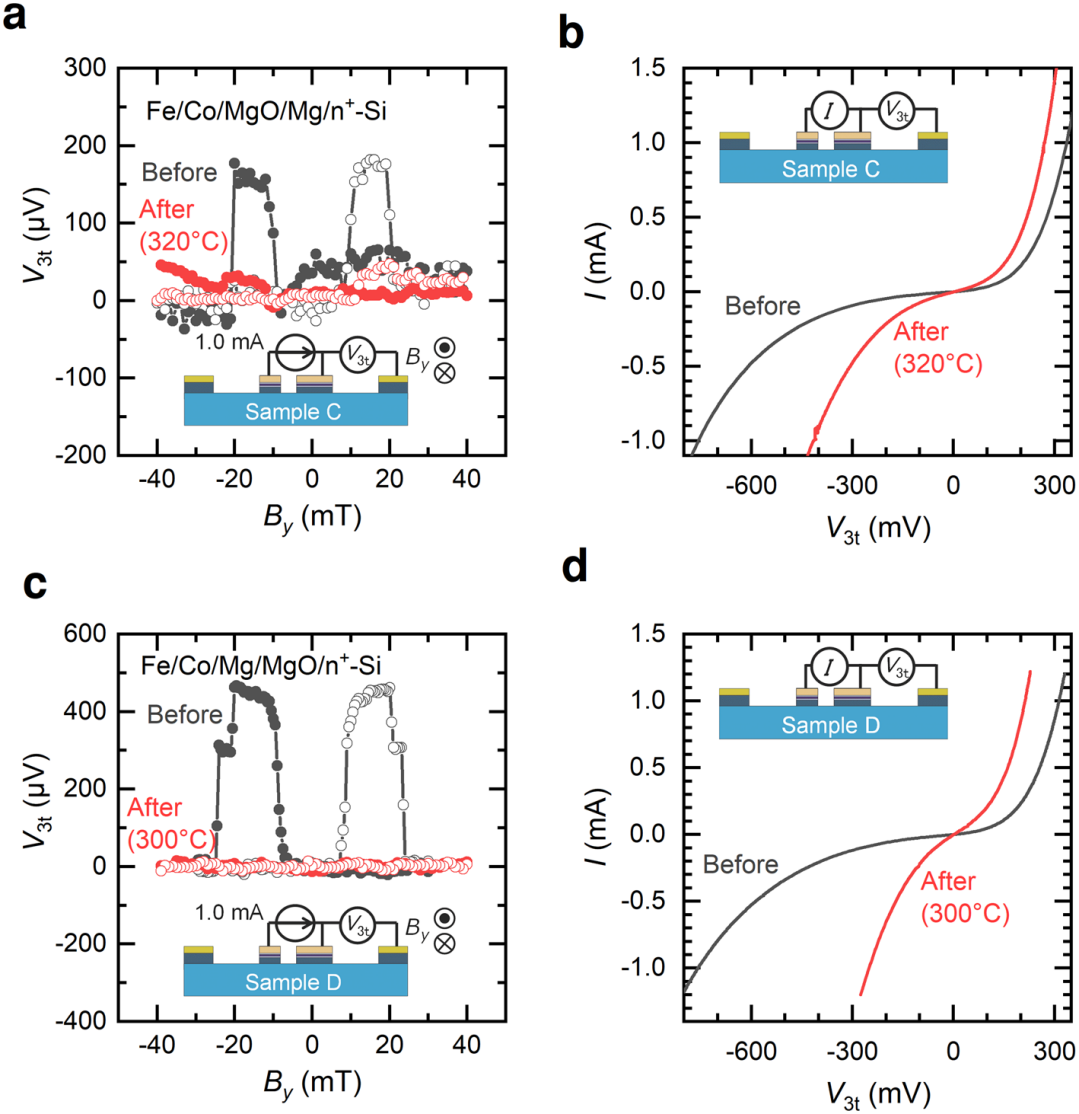
Results of the L3T measurements and *I*–*V* measurements of samples C and D. *L*_ch_ = 2.75 µm, *t* = 100 nm for both samples. The widths of F1 and F2 were 0.5 and 2.0 µm, respectively. (**a**) Results of the L3T measurements of sample C. Black and red plots show the results before and after annealing, respectively. (**b**) Results of the *I*–*V* measurements of sample C. Black and red plots show the results before and after annealing, respectively. (**c**) Results of the L3T measurements of sample D. Black and red plots show the results before and after annealing, respectively. (**d**) Results of the *I–V* measurements of sample D. Black and red plots show the results before and after annealing, respectively.

The thermal tolerance of Mg above the MgO structures is still an open question. The thermal tolerance of NM electrodes is largely improved, which allows for a direct comparison of the thermal tolerance of FM electrodes by the insertion of Mg below/above the MgO layer. Two samples, C and D, with thermally tolerant NM electrodes and FM electrodes with a thin Mg layer below/above the MgO layer, were prepared; the FM electrodes of samples C and D consist of Fe/Co/MgO/Mg and Fe/Co/Mg/MgO, respectively. The annealing temperatures of samples C and D were 320 °C and 300 °C, respectively. Although a clear spin accumulation signal was obtained by L3T measurements even after annealing at 400 °C for sample B, as shown in the inset of Fig. 6a, the spin accumulation signals were strongly suppressed for sample C (Fig. 7a) and not detected for sample D (Fig. 7c) after annealing. The *I*–*V* characteristics of their ferromagnetic electrodes are shown in Fig. 7b, d. The resistance decreased after annealing, which was consistent with the results observed in samples A and B. More importantly, the resistance decreased at much lower temperatures. Because the thermal tolerance of the NM electrodes is higher than 400 °C, the decrease in the thermal tolerance of the device is attributable to the FM electrodes including Mg layer and 0.8 nm-thick MgO tunnelling barrier. The thermal tolerance more than 425 °C was reported in Co_2_FeSi/MgO/Mg/Si structure employing 1.3 nm-thick MgO tunnelling barrier^16,31^, indicating that the thick MgO provides better tolerance. However, we point out that large electrical resistance of the device due to the thick MgO hinders high magnetoresistance ratio. Although some improvements in spin polarization can be reported, the thermal tolerance worsens with the insertion of a Mg layer both below and above the MgO.

## Conclusion

In conclusion, we investigated the degradation mechanism of nondegenerate Si-based LSVs by thermal annealing and found two different mechanisms: Au diffusion from the NM electrode to the FM electrode and degradation of the Mg layer employed for FM electrodes. In the sample using Au/Ta/Al electrodes, interdiffusion of Au and Al atoms occurred, followed by the melting of Au and Si alloy at approximately 350 °C due to the eutectic alloy properties. The Au–Si liquid phase mainly diffused through the AlN/Si interface and finally broke the FM electrodes. By changing the structure from Au/Ta/Al to Au/Ta with a 40-nm-thick Ta layer, the thermal tolerance was improved, and clear spin signals were obtained even after annealing at 400 °C. The insertion of a thin Mg layer below or above the MgO layer was also examined, and their thermal tolerance was less than that without the Mg layer. The maximum thermal tolerance of 400 °C, which was sufficiently high for the post fabrication process of electronic devices was provided by the combination of Au/Ta for NM and Fe/Co/MgO for FM electrodes. The thermally tolerant Si-based LSV certified the greater compatibility of Si-based spin devices with the electronics industry.

## Method

### Sample preparation

Four types of Si-based lateral spin valve devices were prepared. The spin valve structures were fabricated on a silicon-on-insulator substrate with a 100-nm-thick Si(100) layer/200-nm-thick SiO_2_ layer/625-μm-thick Si(100) substrate. Phosphorus (P) atoms were doped into the Si layer by the ion implantation technique to form the n-Si and n^+^-Si (only for sample A) layers. Then, rapid thermal annealing was carried out for their activation. The dopant concentration in the Si channel was confirmed by secondary-ion mass spectrometry, which showed a small distribution perpendicular to the plane in the range of 1 × 10^17^ to 2 × 10^18^ cm^−3^, indicating nondegenerate Si. The dopant concentration of the 20-nm-thick n^+^-Si layer employed to suppress the width of the depletion layer at the ferromagnetic contacts was 5 × 10^19^ cm^−3^. The ferromagnetic contacts F1 and F2 were fabricated by electron beam deposition in an ultrahigh vacuum. Figure 1a shows the layer structures of these electrodes. The deposited materials were Fe (12.4 nm)/CoFe (0.6 nm)/MgO (0.8 nm) for sample A, Fe (12.4 nm)/Co (0.6 nm)/MgO (0.8 nm)/n^+^-Si (20 nm by sputtering) for sample B, Fe (12.4 nm)/Co (0.6 nm)/MgO (0.8 nm)/Mg (0.6 nm)/n^+^-Si (20 nm by sputtering) for sample C and Fe (12.4 nm)/Co (0.6 nm)/Mg (0.6 nm)/MgO (0.8 nm)/n^+^-Si (20 nm by sputtering) for sample D. Electron beam lithography and Ar ion milling were employed to fabricate F1 and F2 and to remove the n^+^-Si layer in the channel region. AlN was deposited on the Si channel as a capping layer. Finally, the NM electrodes were fabricated using electron beam lithography and ion beam deposition. The NM materials of sample A were Au (150 nm)/Ta (5 nm)/Al (40 nm), while Au (150 nm)/Ta (40 nm) was employed for the other samples.

### Thermal annealing

The temperature was increased to the set value within 30 min, kept for 60 min and decreased naturally for 300 min using a Futek K-A102HP furnace. All the annealing processes were conducted in a vacuum (~ 1 × 10^−4^ Pa).

### Non-local four terminal (NL4T) method

A schematic of the current–voltage configuration is shown in the insets of Figs. 1b and 5a. A direct current was applied from N2 to F2. Voltage was measured at F1 and N1 under the magnetic field swept along the *y*-axis in Fig. 1a. Spin polarization *β* was estimated by applying the one-dimensional spin drift–diffusion equation ^32,33^ to spin signal Δ*V*_nl_. In this model, Δ*V*_nl_ is expressed as:

$$ \Delta V_{{{\text{nl}}}} = \frac{{\beta^{2} J}}{{\left\{ {G_{{\text{N}}} + \frac{1}{2}\left( {G_{{{\text{i}}1}} + G_{{{\text{i}}2}} } \right) + \frac{{G_{{{\text{i}}1}} G_{{{\text{i}}2}} }}{{4G_{{\text{N}}} }}} \right\}\exp \left( {\frac{{L_{{{\text{ch}}}} }}{{\lambda_{{{\text{Si}}}} }}} \right) - \frac{{G_{{{\text{i}}1}} G_{{{\text{i}}2}} }}{4}\exp \left( { - \frac{{L_{{{\text{ch}}}} }}{{\lambda_{{{\text{Si}}}} }}} \right)}} , $$

where *J* is the charge current density, *λ*_Si_ is the spin diffusion length of Si, *σ* is the conductivity of Si, *RA*_1_ and *RA*_2_ are the resistance area products of F1 and F2, $G_{{\text{N}}} = \frac{\sigma }{{\lambda_{{{\text{Si}}}} }}$ is the spin conductance of Si and $G_{{{\text{i}}1\left( 2 \right)}} = \frac{1}{{RA_{1\left( 2 \right)} }}$ is the spin conductance of the tunnel barrier of F1(F2). We assumed identical *β* values for F1 and F2 and negligible spin resistance of the ferromagnetic metal. The experimentally measured values of *RA*_1_, *RA*_2_, and *σ* were used for the analysis.

### Local three terminal (L3T) method

A schematic of the current–voltage configuration is shown in the insets of Fig. 6a, c. A direct current of 1.0 mA was applied from F2 to F1. The external magnetic field was swept along the *y*-axis, and the voltage difference was measured at F2 and N2.

## Data Availability

The data that support the findings of this study are available from the corresponding author upon reasonable request.
